# Preparation of Novel Solid Phase Extraction Sorbents for Polycyclic Aromatic Hydrocarbons (PAHs) in Aqueous Media

**DOI:** 10.3390/molecules28166129

**Published:** 2023-08-18

**Authors:** Deogratius T. Maiga, Rose W. Kibechu, Bhekie B. Mamba, Titus A. M. Msagati, Terence T. Phadi

**Affiliations:** 1 Measurement and Control Division, Council for Mineral Technology (MINTEK), Private Bag X3015, Randburg, Johannesburg 2125, South Africa; 2 Institute for Nanotechnology and Water Sustainability, College of Science Engineering and Technology, UNISA Science Campus, University of South Africa, Roodepoort, Johannesburg 1710, South Africa; 3 Department of Chemistry, University of Eswatini, P/Bag 4 Kwaluseni Campus, Kwaluseni 0004, Eswatini; 4 State Key Laboratory of Separation Membranes and Membrane Process, National Center for International Joint Research on Membrane Science and Technology, Tianjin 300387, China

**Keywords:** hybrid stationary phase materials, SPE separation, organic pollutants, gas chromatography coupled with time-of-flight mass spectrometry

## Abstract

In this study, functionalized mesoporous silica was prepared and characterized as a stationary phase using various analytical and solid-state techniques, including a Fourier-transform infrared (FTIR) spectrometer, thermogravimetric analysis, and nitrogen sorption. The results confirmed the successful synthesis of the hybrid stationary phase. The potential of the prepared hybrid mesoporous silica as a solid-phase extraction (SPE) stationary phase for separating and enriching polycyclic aromatic hydrocarbons (PAHs) in both spiked water samples and real water samples was evaluated. The analysis involved extracting the PAHs from the water samples using solid-phase extraction and analyzing the extracts using a two-dimensional gas chromatograph coupled to a time-of-flight mass spectrometer (GC × GC-TOFMS). The synthesized sorbent exhibited outstanding performance in extracting PAHs from both spiked water samples and real water samples. In the spiked water samples, the recoveries of the PAHs ranged from 79.87% to 95.67%, with relative standard deviations (RSDs) ranging from 1.85% to 8.83%. The limits of detection (LOD) for the PAHs were in the range of 0.03 µg/L to 0.04 µg/L, while the limits of quantification (LOQ) ranged from 0.05 µg/L to 3.14 µg/L. Furthermore, all the calibration curves showed linearity, with correlation coefficients (r) above 0.98. Additionally, the results from real water samples indicated that the levels of individual PAH detected ranged from 0.57 to 12.31 µg/L with a total of 44.67 µg/L. These findings demonstrate the effectiveness of the hybrid mesoporous silica as a promising stationary phase for solid-phase extraction and sensitive detection of PAHs in water samples.

## 1. Introduction

As previously defined, mesoporous silica materials are a class of materials which have pore size with diameters ranging between 2 and 50 nm [1]. These materials have garnered attention due to their stability and easy functionalization, making them promising candidates for various applications, including separation [2], adsorption [3,4,5], drug delivery [6], and catalysis [7,8]. The key factors contributing to their wide-ranging applications are their large surface area (ranging from 700 to 1500 m^2^/g) [9], tunable pore size, and chemically accessible silanol groups, which are essential for many applications. One of the well-known types of mesoporous silica is Mobil crystalline material No 41 (MCM-41), which boasts a higher pore volume and exhibits an average hydrophobic nature [10]. The pore size of MCM-41 can be adjusted based on the template used, spanning from 2 to 10 nm [1,11,12]. To enhance their performance, mesoporous silica materials have been functionalized using organic species, leading to improvements in various experiments, such as adsorption chemistry [13], bio-applications [14], electronics [15,16,17], environmental technologies [17], catalysis, sensors [18], chromatography [1,13,19,20] and solid phase extractions [21,22,23,24].

Among the diverse environmental applications, these materials have been employed in the analysis of persistent organic pollutants, specifically polyaromatic hydrocarbons (PAHs). PAHs are organic chemicals that occur naturally in coal, crude oil, and gasoline and pose significant health and environmental risks [21,22]. Due to their carcinogenic and mutagenic properties, it is crucial to establish effective monitoring programs to control their spread [23]. Unfortunately, PAHs are challenging to eliminate from the environment using conventional methods due to their low concentrations, hydrophobicity, and low biodegradability [24]. Thus, extraction and clean-up procedures are essential before determining PAH levels in environmental samples. Solid-phase extraction (SPE) is a widely used sample preparation technique in analytical chemistry, especially for the extraction of trace-level analytes from complex environmental matrices, such as water [20], precipitation (rainwater) [25], and aerosols [26]. Solid-phase extraction offers numerous advantages, such as selectivity, sensitivity, time efficiency, and reproducibility, as well as compatibility with many analytical techniques for the extraction of PAHs, making it a preferred technique for environmental monitoring, research, and regulatory compliance purposes.

In addition to solid-phase extraction (SPE), several advanced extraction techniques have been used for the analysis of polyaromatic hydrocarbons (PAHs) in environmental samples. These techniques offer enhanced sensitivity, selectivity, and efficiency, making them valuable tools for PAH analysis. These techniques include the following. Microwave-assisted extraction (MAE): Microwave-assisted extraction is a rapid and efficient technique that utilizes microwave energy to heat the sample, enhancing the extraction process. It reduces extraction times and solvent consumption while increasing the analyte yield. MAE has been applied to extract PAHs from various matrices, such as soil [27], sediment [28], and food samples [29,30]. Pressurized liquid extraction (PLE): Pressurized liquid extraction, also known as accelerated solvent extraction, employs elevated temperature and pressure to increase the solubility and diffusion of PAHs into the solvent. It allows for high-throughput extraction and can be automated, saving time and labor [31]. Supercritical fluid extraction (SFE): Supercritical fluid extraction uses supercritical fluids (e.g., CO_2_) as the extraction solvent. These fluids possess unique properties between gases and liquids, offering excellent solvating power and selectivity. SFE has been applied to extract PAHs from various environmental samples [32]. Stir bar sorptive extraction (SBSE): SBSE involves a stir bar coated with a polymeric sorbent that is immersed in the sample to extract PAHs. It is a non-destructive and sensitive technique that allows for sample pre-concentration and analysis [33]. Solid-phase microextraction (SPME): Solid-phase microextraction is a solvent-free extraction technique that uses a coated fiber to adsorb PAHs from the sample matrix. It offers simplicity, rapidity, and low sample volume requirements [34]. Dispersive liquid–liquid microextraction (DLLME): DLLME is a microextraction technique that utilizes a mixture of extraction and disperser solvents to form fine droplets. It has been applied to pre-concentrate PAHs from water samples effectively [35]. Magnetic solid-phase extraction (MSPE): MSPE involves the use of magnetic nanoparticles functionalized with a sorbent to extract PAHs. Magnetic separation simplifies the extraction process and reduces the sample preparation time [36]. These advanced extraction techniques offer various advantages over traditional methods, including reduced sample preparation time, lower solvent consumption, and higher analyte recovery. Nevertheless, the choice of extraction technique depends on the specific application and the properties of the target PAHs in the environmental sample.

In this research, we focus on the synthesis and surface modification of mesoporous silica materials to develop novel solid-phase extraction sorbents for the analysis of PAHs. The synthesized materials were subjected to thorough characterization to assess their chemical properties. Subsequently, the hybrid mesoporous materials were packed into empty SPE cartridges and used for the separation and enrichment of PAH compounds in both spiked water samples and real water samples. The efficiency of the packed SPE cartridges in pre-concentrating PAHs, including acenaphthene, fluorene, fluoranthene, pyrene, benzo[k]fluoranthene, perylene, indeno[1,2,3-cd]fluoranthene, and benzo[ghi]perylene from both spiked water samples and real water samples was investigated using GC × GC-TOFMS. The results revealed that the synthesized hybrid material/sorbent exhibited exceptional stability, high capacity, a large surface area, and enhanced selectivity for PAHs compared to other reported sorbents [20,28,29].

## 2. Results and Discussion

### 2.1. FTIR Measurements

**Figure 1:**
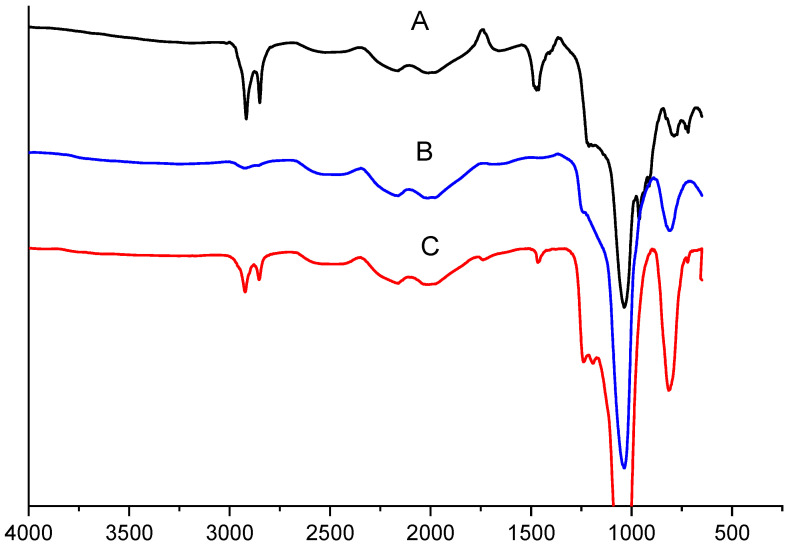
FTIR spectra for (A) as-synthesized silica, (B) mesoporous silica, and (C) modified silica.

The FTIR spectra of the silica materials are depicted in Figure 1(A–C). In Figure 1(A), peaks are observed around 2915.9 cm^−1^ and 2852.6 cm^−1^, along with the Si–O–Si stretching band occurring between 1240 cm^−1^ and 1030 cm^−1^, which is characterized as the fingerprint region of the parent silica [10]. Figure 1(B) shows the disappearance of the peaks around 2915.9 cm^−1^ and 2852.6 cm^−1^. This indicates that the calcination process was successful, and the organic surfactant was completely removed from the parent silica [19]. Finally, in Figure 1(C), new emerging peaks around 2924.8 cm^−1^ and 2849.3 cm^−1^ are observed, which can be attributed to C–H stretching of methyl/alkyl groups. The occurrence of these new peaks indicates the successful chemical interaction between the silanol groups on the surface of the mesoporous silica and the organic groups [10,37].

### 2.2. Thermogravimetric Analysis

**Figure 2:**
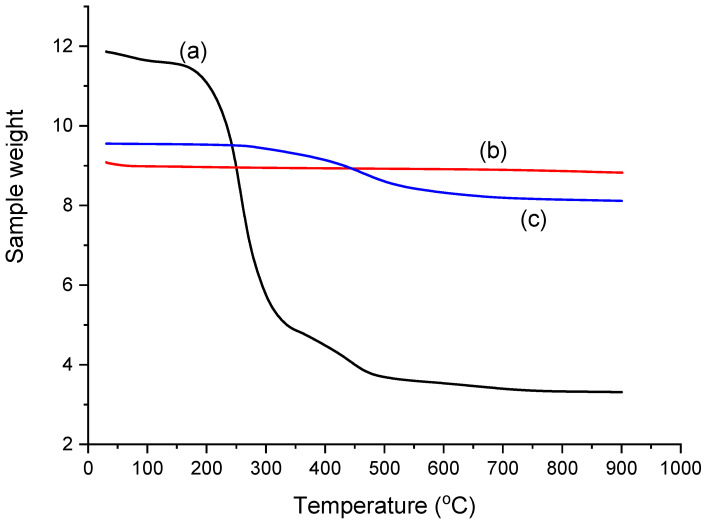
TGA curve of (a) As-synthesized mesoporous silica, (b) mesoporous silica, and (c) mesoporous silica modified with C18 alkyl groups.

Figure 2(a) describes the TGA curves for the as-synthesized mesoporous silica in different phases of weight loss. The first phase of weight loss was observed between 50 and 150 °C, where approximately 3% was lost due to the removal of adsorbed water on the materials. However, the second phase observed in the range of 150–330 °C, where there was approximately 54.7% weight loss, might be assigned to the removal of organic groups present in the mesoporous silica. These organic groups might be residues from the synthesis process or other functional groups attached to the silica surface. The third phase of weight loss at the temperature between 330 and 550 °C, with approximately 12.06% weight loss, may be attributed to the decomposition of the surfactant. Above 550 °C, no further weight loss was observed, indicating that the surfactant had been completely removed, and the material was then stable at higher temperatures [37,38].

Figure 2(b) shows TGA curves of mesoporous silica. Below 100 °C, it was observed that there was no significant difference in weight loss. However, even at temperatures above 100 °C, no weight loss was observed still, showing that the material was stable throughout the heating temperature.

Figure 2(c) depicts TGA profiles of modified mesoporous with C18 alkyl groups under a nitrogen atmosphere. At temperatures between 250 and 650 °C, very steady degradation was observed, and approximately 13.82% weight loss was recorded. This may be attributed to the removal of the C18 alkyl groups and HMDS incorporated in the mesoporous silica. Above 750 °C, no weight loss was observed, showing that the material is now stable at higher heating temperatures. This suggests that the C18 alkyl groups and HMDS had been fully removed from the modified mesoporous silica, and the material had reached a stable state.

### 2.3. Nitrogen Sorption Studies

#### 2.3.1. BET Surface Area

Table 1 presents the results for BET surface area analysis for silica materials before and after surface modifications. The results show a higher surface area for calcined mesoporous silica (1321.0274 m^2^/g), as expected according to previous reports [19,20,39]. However, the surface areas for mesoporous silica modified with C18 alkyl groups and end-capping with HMDS decreased to 1269.4379 and 991.8470 m^2^/g, respectively. This is due to the replacement of the silanol groups on mesoporous silica with the organic groups. This also proves that the functionalization of the mesoporous silica with the organic groups was successful. However, after silylation and end-capping processes, the materials still have higher surface areas, which have greater importance in adsorption chemistry.

#### 2.3.2. Pore Size Distribution

Barett, Joyner, and Halenda (BJH) methods were utilized for the evaluation of the pore diameter and pore size distribution of the materials, as shown in Table 1, which indicates that the resulting pore diameter of the materials corresponds to the previous literature [19,20,40]. The result shows that there is a reduction in pore diameter as the C18 alkyl groups and HMDS are incorporated in the mesoporous silica. However, this proves that the alkyl chains were successfully attached to the mesoporous silica materials.

### 2.4. Method Performance and Validation

The developed method was assessed for its practical applicability by investigating the figures of merit of the method validation using PAHs spiked in deionized water. The summarized results are presented in Table 2. The linearity of the method was found to be good, with determination coefficients (R-squared) ranging from 0.98 to 0.99. This indicates that the calibration curves for the analytes showed a strong correlation between the concentration of PAHs and their corresponding responses. In terms of accuracy, a good recovery was achieved for the spiked PAHs at a concentration level of 4 µg/L. The recovery values ranged from 79.87% to 95.67%, indicating that the method was capable of accurately determining the PAH concentrations in the water samples. The precision of the method was evaluated by calculating the %RSD (relative standard deviation) based on the repeatability of results from the recovery experiments. The obtained precision was satisfactory, with %RSD values ranging from 1.85% to 8.83%. This indicates that the method produced consistent and reproducible results. To assess the sensitivity of the method, the LOD (limit of detection) and LOQ (limit of quantification) of the analytes were determined following the procedure described in Section 3.9. The LOD and LOQ values were found to be low, indicating that the method had sufficient sensitivity to detect and quantify the amounts of PAHs in water.

The comparison of different methods for the determination of PAHs is presented in Table 3. The SPE method [41] exhibited low LOD and higher recovery but had high LOQ. The SPE method [22] showed a higher percentage recovery but exhibited high LOD. The LLE method [42] exhibited low LOQ but had a lower percentage recovery and comparatively required high sample volume. The SBSE method [33] exhibited low LOD and low LOQ but showed a relatively lower percentage recovery. The SPE method [22] revealed low LOD and low LOQ but showed a relatively lower percentage recovery. The DLLME method [43] exhibited higher LOQ. The proposed method SPE- GC × GC-TOF-MS exhibited low LOD, low LOQ, good precision, and higher recovery better than or equivalent to those revised methods.

### 2.5. The GCxGC-TOF-MS Chromatograms of Acp, Flu, FL, Pyr, BkF, Per, InF, and BghiP

**Figure 3:**
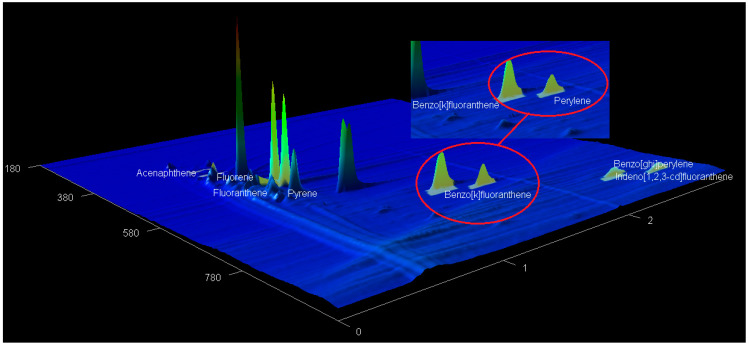
Typical 2D chromatogram for extracted PAHs from spiked water sample obtained by 2D GC × GC-TOFMS.

**Figure 4:**
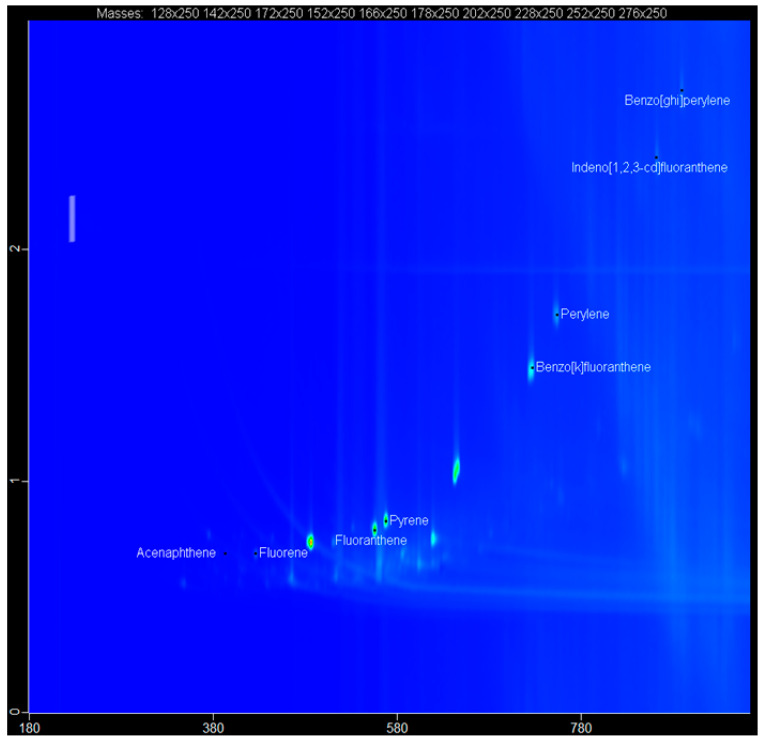
Contour plot of a GC × GG-TOFMS chromatogram for extracted PAHs from spiked water sample.

**Figure 5:**
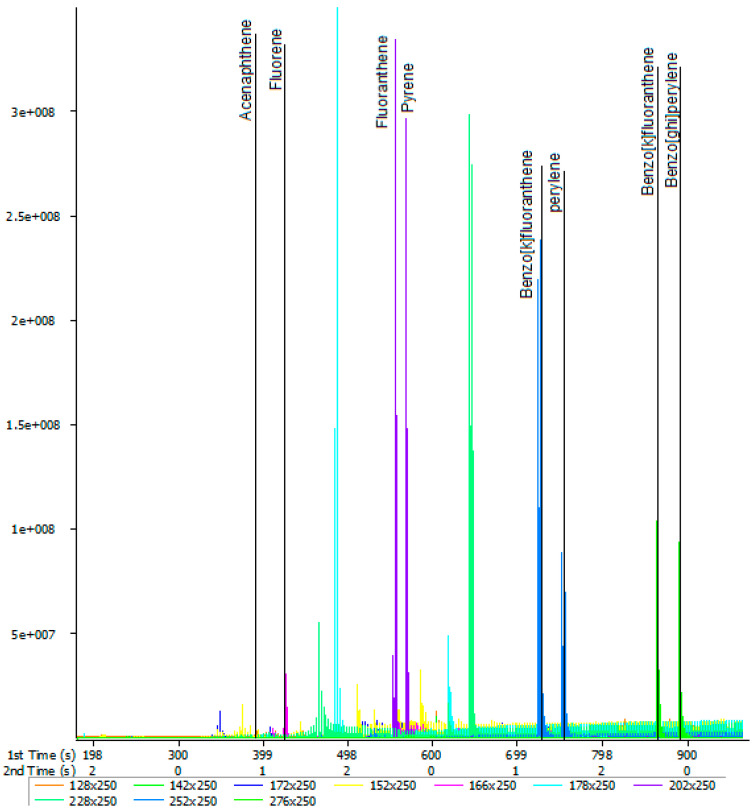
Chromatogram for extracted PAHs from spiked water sample obtained by 2D GC × GG-TOFMS.

The targeted compound (acenaphthene (Acp), fluorene (Flu), fluoranthene (FL), pyrene (Pyr), benzo[k]fluoranthene (BkF), perylene (Per), indeno[1,2,3-cd]fluoranthene (InF), and benzo[ghi]perylene (BghiP)) from the spiked water samples were identified based on the retention time, accurate mass measurement of the molecular ion and by automated mass spectral library searches with GC × GC-TOF-MS (Table 1). All the PAH compounds obtained from GC × GC-TOF-MS are presented in Figure 3, Figure 4 and Figure 5.

### 2.6. Applications on Real Water Samples

The method was applied to analyze actual water samples collected from the Buffalo River Estuary in the Eastern Cape Province of South Africa. All eight PAHs were successfully detected, with individual PAH concentrations falling within the range of 0.57–12.31 µg/L and having a total concentration of 44.67 µg/L, as presented in Table 4 below. This demonstrates the method’s capability to identify a diverse range of PAH compounds in water samples. Additionally, the results indicate that the method is sensitive enough to measure low concentrations of these compounds in water. Furthermore, the total concentration of PAHs in the water samples provides a comprehensive measure of the PAH pollution in the Buffalo River Estuary. However, it is important to note that the water samples analyzed may not fully represent the overall water quality in the area. To ensure accurate and reliable results, proper sampling protocols and randomization are crucial in order to reflect the actual conditions of the estuary.

## 3. Experimental Section

### 3.1. Standard and Reagents

The standards and reagents used in the experiment were of high quality and analytical grade. The High-Performance Liquid Chromatography (HPLC) and Gas Chromatography (GC) grade solvents, acetonitrile, hexane, toluene, acetone, pentane, and ethanol, were supplied by Sigma-Aldrich in Johannesburg, South Africa. The specific compounds tetraethyl orthosilicate (TEOS), 1,1,3,3,3-hexamethyldisilazane (HMDS), cetyltrimethylammonium bromide (CTAB), and n-octadecyltrimethoxysilane were supplied by Sigma-Aldrich in Johannesburg, South Africa. Polyaromatic Hydrocarbons (PAHs) standards acenaphthene (Acp), fluorene (Flu), fluoranthene (FL), pyrene (Pyr), benzo[k]fluoranthene (BkF), perylene (Per), indeno[1,2,3-cd]fluoranthene (InF), and benzo[ghi]perylene (BghiP) were also supplied by Sigma-Aldrich in Johannesburg, South Africa. The other chemicals, such as sodium hydroxide pellets, were obtained from Rochelle Chemicals in Johannesburg, South Africa.

### 3.2. Sampling

The water samples were collected from the Buffalo River Estuary in the Eastern Cape Province of South Africa. The samples were collected at a depth of 30 to 60 cm below the water surface and stored in pre-cleaned amber glass bottles with Teflon tops. After collection, the water samples were transported in an ice box to the laboratory, filtered through a 0.45 µm filter, and stored in a fridge at 4 °C before analysis.

### 3.3. Synthesis and Functionalization

#### 3.3.1. Synthesis of Silica Materials

The new materials were synthesized following the modification of the earlier described procedures [1,10,19,30,31,44]. The modification involved changes in experimental parameters from the previous procedure to improve the properties of the synthesized materials. The materials were prepared with a molar ratio of 1:0.25:0.1:20 for TEOS:NaOH:CTAB:H_2_O, respectively. After the addition of these materials to the reaction vial, the suspension formed was stirred at an ambient temperature for 30 min and then heated at 115 °C for 20 h. Then, the white precipitate materials were formed, collected, filtered, and washed with deionized water before being dried overnight at 80 °C. Then, the prepared materials were calcined for 8 h at 550 °C to remove the template.

#### 3.3.2. Functionalization of Silica Materials

The synthesized mesoporous materials were modified following the previously reported methods [30,31,32,33] with some modifications. Functionalization was performed by a grafting technique using n-octadecyltrimethoxysilane and HMDS as functionalization agents. The method used for the grafting of mesoporous silica materials was as follows: dried silica materials (1 g) were added onto 50 mL of dry toluene followed by the addition of 6 g of n-octadecyl trichlorosilane while stirring. The suspension was then stirred and refluxed at 120 °C for 48 h, followed by the addition of 3 g of HMDS for end-capping. The suspension was continuously stirred and refluxed for 24 h. After attaining ambient temperature, the modified materials were filtered and washed using a series of organic solvents and finally dried overnight at 80 °C.

### 3.4. Characterization of Silica Materials

The silica materials were characterized by a Fourier-transform infrared (FTIR) spectrometer to establish the functional groups created, and a Thermogravimetric analyzer (TGA) was employed to evaluate the thermal stability of the materials, while nitrogen sorption, also known as the Brunauer–Emmett–Teller (BET) method, was used to provide a measure of their pore diameter and surface areas.

#### 3.4.1. FTIR Measurements

During the FTIR experiments to establish the functional groups of the prepared silica materials, pellets were prepared in KBr, at a ratio of 20:1, under vacuum using a Hydraulic Press (International Crystal laboratories, Garfield, NJ, USA). The Fourier-transform infrared (FTIR) spectrometer analysis was performed using a Perkin Elmer 100 FTIR instrument (Shelton, WA, USA), covering a spectrum from 650 to 4000 cm^−1^.

#### 3.4.2. Thermogravimetric Analyser (TGA)

The thermal stability of the silica materials and physicochemical properties of the surfactant degradation of the materials were monitored by using Pyris TGA-Perkin Elmer analyzer (Shelton, WA, USA) under a nitrogen gas flow (20 mL/min). The instrument temperature was programmed from 30 to 900 °C, and ramping rate was set at 10 °C/min.

#### 3.4.3. Nitrogen Sorption Studies

Nitrogen adsorption–desorption isotherm analysis was carried out using Micromeritics ASAP 2010 (Norcross, GA, USA). Silica materials weighing between 0.2 and 0.3 g were degassed at 120 °C and analyzed. The BET method was employed to measure the surface area at a relative pressure of 0.3, while the BJH method was used to establish the pore size of the materials.

### 3.5. Preparation of SPE Cartridges

The lab-packed SPE cartridges were created using synthesized C18 materials and 6 mL empty cartridges. Methanol was used as the solvent during the process. To pack the cartridges, the slurry technique was employed [21,34,35]. Initially, the empty cartridges were mounted on the SPE manifold from Supelco, USA, and connected to a vacuum pump. Then, a slurry containing 400 mg of silica materials was carefully introduced into each empty cartridge. The packed cartridges were further washed with deionized water and vacuum-dried to complete the preparation process.

### 3.6. Preparation of Calibration Solutions

The calibration standard solutions were prepared to analyze PAHs, including acenaphthene (Acp), fluorene (Flu), fluoranthene (FL), pyrene (Pyr), benzo[k]fluoranthene (BkF), perylene (Per), indeno[1,2,3-cd]fluoranthene (InF), and benzo[ghi]perylene (BghiP). Acetonitrile was used as the solvent for dissolving the PAHs, resulting in a series of calibration standard solutions with concentrations ranging from 0.01 µg/L to 5 µg/L. These calibration standards served as the basis for establishing a calibration curve to quantify PAHs during the sample analysis.

### 3.7. SPE Extraction Procedure

Before applying the samples, the laboratory-prepared solid-phase extraction (SPE) cartridges underwent a conditioning process with methanol (10 mL) followed by deionized water (5 mL). After that, the samples (100 mL) were loaded onto the cartridges, and a wash with deionized water (5 mL) was performed to remove unwanted compounds. The cartridges were then dried using a vacuum, and the analytes were eluted with acetonitrile (5 mL).

Subsequently, the eluate was dried further using nitrogen gas under a water bath at 30 °C. Finally, the dried eluate was reconstituted with acetonitrile (1 mL) to prepare for injection into the GC × GC-TOFMS for analysis. Each experiment was conducted in triplicates to ensure the reliability of the results [21,36,45]. The reconstitution step played a crucial role in pre-concentrating the eluate, resulting in improved sensitivity for the subsequent analysis. If we were to elute the solid-phase extraction (SPE) cartridge directly with only 1 mL of acetonitrile, it would not be sufficient to release an adequate amount of the analyte.

#### 3.7.1. Extraction of Spiked Water Samples

To validate the method, a 100 mL aliquot of water spiked with 4 µg/L of polycyclic aromatic hydrocarbon (PAH), namely acenaphthene (Acp), fluorene (Flu), fluoranthene (FL), pyrene (Pyr), benzo[k]fluoranthene (BkF), perylene (Per), indeno[1,2,3-cd]fluoranthene (InF), and benzo[ghi]perylene (BghiP), was subjected to extraction. The extraction process was carried out using the specially prepared solid-phase extraction (SPE) cartridge, following the procedure outlined in Section 3.7.

#### 3.7.2. Applications on Real Water Samples

The actual water samples collected from the Buffalo River Estuary in the Eastern Cape Province of South Africa were extracted using the extraction procedure outlined in Section 3.7.

### 3.8. Instrument and Analytical Conditions

The Agilent© 7890A Gas Chromatograph, LECO© GC × GC-TOF-MS system (St Joseph, MI, USA) was utilized for the detection and quantification of PAHs. The primary column used for the separation of the PAHs was the Rxi-5SilMS, and the secondary column used was the Rxi-17SilMS. The sample volume of 1 µL was injected into the GC × GC-TOF-MS with a split ratio of 1:10 and a flow rate of 1.4 mL/min. The oven temperature was initially programmed at 50 °C, held for 0.5 min, and thereafter, ramped up to 290 °C at 25 °C/min and to 320 °C at 5 °C/min. The transfer line and inlet temperatures of 290 °C and 300 °C, respectively, were utilized. The total run time for analysis was 16 min [22,23]. The calibration curves were obtained using GC × GC-TOF-MS in the selected ion mass (SIM) acquisition mode. Prior to sample injection, the syringe was cleaned three times with n-hexane. Data processing was performed on 2D chromatograms using LECO ChromaTOF software (version 4.50.8.0). 

Table 5 shows the method parameters used in this work for the determination of PAHs from spiked water samples.

### 3.9. Determination of Limits of Quantification (LOQ), Limits of Detection (LOD) and Linearity of PAHs

Linear regression analysis was used to determine the *LOD* and *LQD* for PAHs based on GC × GC-TOF-MS linear calibration curves for each PAH. It was assumed from the obtained linear calibration curves for each PAH that the GC × GC-TOF-MS response matrix Y was linearly related to the descriptor matrix X for a limited range of concentrations. The *LOD* and *LOQ* for each PAH were thus determined based on the residual standard deviation of a regression line or the standard deviation (*SD*) of the y-intercepts of the regression line of the calibration curve and the sensitivity or slope of the regression line, as shown in Equations (1) and (2) [46].

(1) $$LOD=3.3\times \frac{SD}{Slope}$$

(2) $$LOQ=10\times \frac{SD}{Slope}$$

## 4. Conclusions

In conclusion, this study successfully synthesized stationary phase materials with higher surface areas, which significantly enhanced the adsorption capabilities for the extraction of various PAHs from both spiked water samples and real water samples. The advanced synthesis of these stationary phase materials has paved the way for more efficient and simultaneous extraction of PAHs, enabling a more comprehensive analysis. The utilization of gas chromatography coupled with comprehensive two-dimensional gas chromatography and time-of-flight mass spectrometry (GC × GC-TOFMS) for quantification has further enhanced the analytical capabilities of this approach. This advanced analytical technique enables precise identification and quantification of PAHs in complex samples, contributing to a more comprehensive understanding of their presence and potential environmental impact. Overall, this study represents a significant advancement in the field of PAH analysis, offering a robust and efficient method for the simultaneous extraction and determination of these compounds from water samples. The combination of novel stationary phase materials and state-of-the-art analytical techniques opens up new avenues for environmental monitoring, risk assessment, and ensuring the safety of water resources.

## Figures and Tables

**Table 1:** Nitrogen sorption characteristics for silica materials before and after surface modification.

Materials	S_BET_ (m^2^/g)	BJH Dpore (nm)
Mesoporous silica	1321.0	3.8
Mesoporous silica modified with n-octadecyltrimethoxysilane	1269.4	2.8
Mesoporous silica modified and end-capped with HMDS	991.8	2.3

SBET: BET surface area; Dpore: pore diameter.

**Table 2:** Analytical parameters for the determination of PAHs.

Compound	Linearity (R^2^)	%RSD (*n* = 3)	Recovery (%) (*n* = 3)	LOD (µg/L)	LOQ (µg/L)
Acp	0.99	8.72	81.53	0.19	0.58
Flu	0.98	6.53	84.53	1.04	3.14
FL	0.98	2.92	80.80	0.31	0.94
Pyr	0.99	8.83	91.07	0.57	1.72
BkF	0.98	5.27	82.47	0.51	1.53
Per	0.98	8.39	95.67	0.04	0.14
InF	0.99	6.52	89.73	0.02	0.05
BghiP	0.99	1.85	79.87	0.75	2.26

**Table 3:** Comparison between proposed method and other extraction methods for the determination of PAHs.

Preconcentration Technique	Analytical Technique	Sample Volume (mL)	LOD (µg/L)	LOQ (µg/L)	RSD (%)	Recovery (%)	Reference
SPE	HPLC-Flu ^a^	50	<0.02	0.7–56.2	2–5	67–99	[41]
SPE	GC-MS	10	50–500	NR ^b^	1.56–4.47	87.3–97.4	[20]
LLE ^c^	HPLC-Flu	250	NR	0.1–4.4	NR	51–104	[42]
SBSE ^d^	HPLC-Flu	39.5	0.0005–0.007	0.001–0.022	<9	43–57	[33]
SPE	GC-FID ^e^	105	0.01–0.04	0.05–0.16	3.8–22.2	30.9–119	[23]
DLLME ^f^	GC-FID	5	0.007–0.030	23.3–100	1.4–10.2	60–111	[43]
SPE	GCxGC-TOF-MS	10	0.03–0.04	0.05–3.14	1.85–8.83	80–96	This work

^a^ Liquid chromatography (LC) with fluorescence detection, ^b^ Not reported, ^c^ liquid–liquid extraction, ^d^ Stir bar sorptive extraction, ^e^ gas chromatography with flame ionization detection, and ^f^ dispersive liquid–liquid microextraction.

**Table 4:** PAH levels in real water samples.

Compound	Abbreviation	Surface Water (µg/L)
Acenaphthene	Acp	8.57
Fluorene	Flu	4.28
Fluoranthene	FL	1.48
Pyrene	Pyr	3.13
Benzo[k]fluoranthene	BkF	5.54
Perylene	Per	0.57
Indeno[1,2,3-cd]fluoranthene	InF	8.77
Benzo[ghi]perylene	BghiP	12.31
Σ PAHs		44.67
Σ PAHs = sum of polycyclic aromatic hydrocarbons		

**Table 5:** GC × GC-TOF-MS parameters for the determination of PAHs.

Compound	Abbreviation	Retention Time (min)	Quantified Masses
Acenaphthene	Acp	6.40	154
Fluorene	Flu	7.10	166
Fluoranthene	FL	9.25	202
Pyrene	Pyr	9.45	202
Benzo[k]fluoranthene	BkF	12.10	252
Perylene	Per	12.55	252
Indeno[1,2,3-cd]fluoranthene	InF	14.35	276
Benzo[ghi]perylene	BghiP	14.80	276

## Data Availability

The datasets used and/or analyzed during the current study are available from the corresponding author upon reasonable request.

## Sample Availability

Samples of the compounds are not available from the authors.
